# Salivary Myeloperoxidase, Assessed by 3,3′-Diaminobenzidine Colorimetry, Can Differentiate Periodontal Patients from Nonperiodontal Subjects

**DOI:** 10.1155/2016/7517928

**Published:** 2016-05-05

**Authors:** Supaporn Klangprapan, Ponlatham Chaiyarit, Doosadee Hormdee, Amonrujee Kampichai, Tueanjit Khampitak, Jureerut Daduang, Ratree Tavichakorntrakool, Bhinyo Panijpan, Patcharee Boonsiri

**Affiliations:** ^1^Department of Biochemistry, Faculty of Medicine, Khon Kaen University, Khon Kaen 40002, Thailand; ^2^Department of Oral Diagnosis, Faculty of Dentistry, Khon Kaen University, Khon Kaen 40002, Thailand; ^3^Research Group of Chronic Inflammatory Oral Diseases and Systemic Diseases Associated with Oral Health, Khon Kaen University, Khon Kaen 40002, Thailand; ^4^Department of Periodontology, Faculty of Dentistry, Khon Kaen University, Khon Kaen 40002, Thailand; ^5^Dental Department, Fang Hospital, Fang District, Chiangmai 50110, Thailand; ^6^Department of Clinical Chemistry, Faculty of Associated Medical Sciences, Khon Kaen University, Khon Kaen 40002, Thailand; ^7^Centre for Research and Development of Medical Diagnostic Laboratories, Khon Kaen University, Khon Kaen 40002, Thailand; ^8^Department of Clinical Microbiology, Faculty of Associated Medical Sciences, Khon Kaen University, Khon Kaen 40002, Thailand; ^9^Faculty of Science, Mahidol University, Bangkok 10400, Thailand

## Abstract

Periodontal diseases, which result from inflammation of tooth supporting tissues, are highly prevalent worldwide. Myeloperoxidase (MPO), from certain white blood cells in saliva, is a biomarker for inflammation. We report our study on the salivary MPO activity and its association with severity of periodontal diseases among Thai patients. Periodontally healthy subjects (*n* = 11) and gingivitis (*n* = 32) and periodontitis patients (*n* = 19) were enrolled. Assessments of clinically periodontal parameters were reported as percentages for gingival bleeding index (GI) and bleeding on probing (BOP), whereas pocket depth (PD) and clinical attachment loss (CAL) were measured in millimeters and then made to index scores. Salivary MPO activity was measured by colorimetry using 3,3′-diaminobenzidine as substrate. The results showed that salivary MPO activity in periodontitis patients was significantly higher than in healthy subjects (*p* = 0.003) and higher than in gingivitis patients (*p* = 0.059). No difference was found between gingivitis and healthy groups (*p* = 0.181). Significant correlations were observed (*p* < 0.01) between salivary MPO activity and GI (*r* = 0.632, *p* < 0.001), BOP (*r* = 0.599, *p* < 0.001), PD (*r* = 0.179, *p* = 0.164), and CAL (*r* = 0.357, *p* = 0.004) index scores. Sensitivity (94.12%), specificity (54.55%), and positive (90.57%) and negative (66.67%) predictive values indicate that salivary MPO activity has potential use as a screening marker for oral health of the Thai community.

## 1. Introduction

Periodontal diseases are bacterial infected diseases, which are highly prevalent worldwide [1, 2]. Periodontal status in the oral cavity is classified into healthy and periodontal diseases. The periodontal diseases include gingivitis and periodontitis [1]. Gingivitis affects only the gingival tissues without alveolar bone destruction, whereas periodontitis leads to alveolar bone destruction [1]. In Thailand, the seventh national oral health survey conducted in the year 2012 reported that children aged 12 and 15 had gingivitis (50.30 and 53.60%, resp.) and adults (age 35–44) and the elderly (age 60–74) had periodontal diseases for 85.90% and 88.50%, respectively [3].

Periodontal diseases are associated with the systemic diseases such as diabetes mellitus [4], ischemic heart disease, and acute coronary syndromes [5, 6]. Therefore, early detection of periodontal diseases is required for successful treatment and to reduce disease severity and complications.

Several studies reported the correlation of periodontal diseases and some biomarkers including IFN-*γ*, IL-10, IL-17, IL-1beta, lactoferrin, and myeloperoxidase (MPO) [7, 8]. MPO is released from azurophilic granules of polymorphonuclear cells or neutrophils to catalyze the formation of bactericidal compounds such as hypochlorous acid (HOCl) [9–11]. Increased MPO activity in gingival crevicular fluid (GCF) from patients with periodontal diseases has been reported [12–14]. The objective of this study was to determine MPO activity in saliva and its association with severity of periodontal diseases among Thai patients.

## 2. Materials and Methods

### 2.1. Chemicals and Reagents

3,3′-Diaminobenzidine tetrahydrochloride (DAB) was obtained from Sigma, USA. Hydrogen peroxide (H_2_O_2_) was purchased from Merck, Germany. The other chemicals used were all of analytical grade.

### 2.2. Study Population and Selection Criteria

Sixty-two systemically healthy Thai individuals (24 males and 38 females), aged 19–66, who checked up their oral health at Faculty of Dentistry, Khon Kaen University, Thailand, during October-November 2013, were included in the present study. According to clinical classification of periodontal diseases by Armitage [1], assessment of periodontal parameters including gingival bleeding index (GI) and bleeding on probing (BOP) [15] and pocket depth (PD) and clinical attachment loss (CAL) [16] was performed by one examiner. A probe UNC-15 (Hu-Friedy, Chicago, IL) was employed during monitoring. Subjects were divided into 3 groups: periodontally healthy individuals as a control group (*n* = 11); gingivitis patients (*n* = 32); and periodontitis patients (*n* = 19). Inclusion criteria for periodontally healthy individuals were as follows: any individual who had at least 10 remaining teeth, GI ≤ 20%, BOP ≤ 20% of sites, no PD formation, and no CAL. For gingivitis patients, the inclusion criteria were any individual who had at least 10 remaining teeth, GI > 20%, BOP > 20% of sites, PD ≤ 4 mm, and CAL ≤ 1 mm. Chronic periodontitis patients were included according to the following criteria: any individual who had at least 10 remaining teeth, GI > 20%, BOP > 20% of sites, PD > 4 mm, and CAL > 1 mm. Upon clinical examination, assessment of PD was inferred to the scores as follows: 1: less than 4 mm; 2: 4 to 6 mm; 3: more than 6 mm [17]. Assessment of CAL was inferred to the scores as follows: 0: no clinical attachment loss; 1: less than 2 mm; 2: 2 to 4 mm; 3: more than 4 mm [18]. The PD index score in each individual was established as follows: each PD score was multiplied by number of sites demonstrating PD score. The sum of multiplied PD scores was divided by the totally measured PD sites. The CAL index score in each individual was established as follows: each CAL score was multiplied by number of sites demonstrating CAL score. The sum of multiplied CAL scores was divided by the totally measured CAL sites. Those with history of any systemic disease, smoking, current pregnancy or lactation, periodontal therapy or use of antibiotics, or mouth rinse in the previous 3 months were excluded. This study was approved by the human ethics committee, Khon Kaen University (HE551372).

### 2.3. Saliva Collection

Unstimulated whole saliva samples were collected in the morning (09.00–11.00 am) at the dental clinic, Faculty of Dentistry, Khon Kaen University. No food was allowed for the subjects at least 90 minutes before collection of saliva. Each individual was instructed to rinse the mouth thoroughly with water, followed by expectorating whole saliva into a 50 mL centrifuge tube. A final saliva volume of 3 to 5 mL was obtained from each subject and the sample kept in an icebox. Measurement of salivary pH and MPO activity was performed immediately.

### 2.4. Colorimetric Assay of Salivary MPO Activity

Salivary MPO activity was measured by using DAB as substrate according to a modified method of Herzog and Fahimi [19]. Each saliva sample (100 *μ*L) was pipetted into 1 mL of the 0.5 mM DAB solution (0.9 g DAB in 50 mL of 0.1 M potassium dihydrogenphosphate pH 4.5). Fifty *μ*L of 6 mM H_2_O_2_ was added to initiate the reaction. After incubation at room temperature for 20 minutes, 20 *μ*L of 0.1 mM sodium azide was added to stop the reaction. Absorbances were measured at 465 nm (Genesys 20 Thermo Scientific, USA).

### 2.5. Statistical Analysis

Statistical analyses were performed using SPSS program (version 11.5). The Kolmogorov-Smirnov test and the Shapiro-Wilk test were used to assess the distribution of the investigated data. Comparisons of salivary MPO activity among the three groups (healthy, gingivitis, and periodontitis) were analyzed using one-way ANOVA. Pearson's correlation coefficient was calculated to determine the correlation between salivary MPO activity and severity of periodontal diseases. Two-tailed *p* < 0.05 was considered statistically significant. The sensitivity, specificity, positive predictive value, and negative predictive value were analyzed using STATA program (version 10.1). The cut-off point was obtained according to Youden's index [20].

## 3. Results

### 3.1. Characteristics of Study Subjects

Demographic characteristics, salivary pH, and periodontal clinical parameters of the study subjects are provided in Table 1. Kolmogorov-Smirnov test and the Shapiro-Wilk test showed that age, CAL index scores, and PD index scores were not normally distributed. The healthy and gingivitis subjects were significantly younger than periodontitis patients. All periodontal clinical parameters (GI, BOP, PD, and CAL) in periodontal disease patients were significantly higher than those in the control group.

### 3.2. Measurement of Salivary MPO Activity

**Figure 1:**
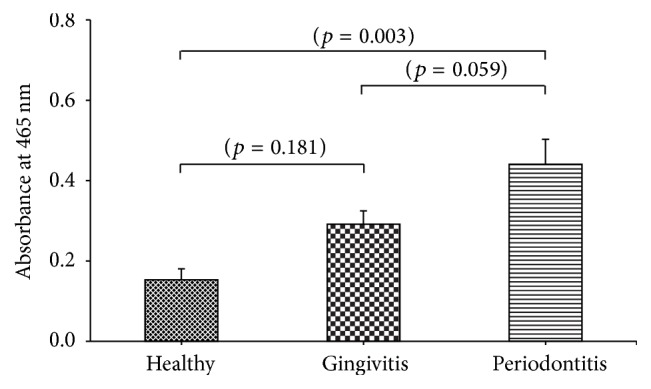
Comparison of salivary MPO activity among periodontally healthy individuals (*n* = 11), gingivitis patients (*n* = 32), and periodontitis patients (*n* = 19).

**Figure 2:**
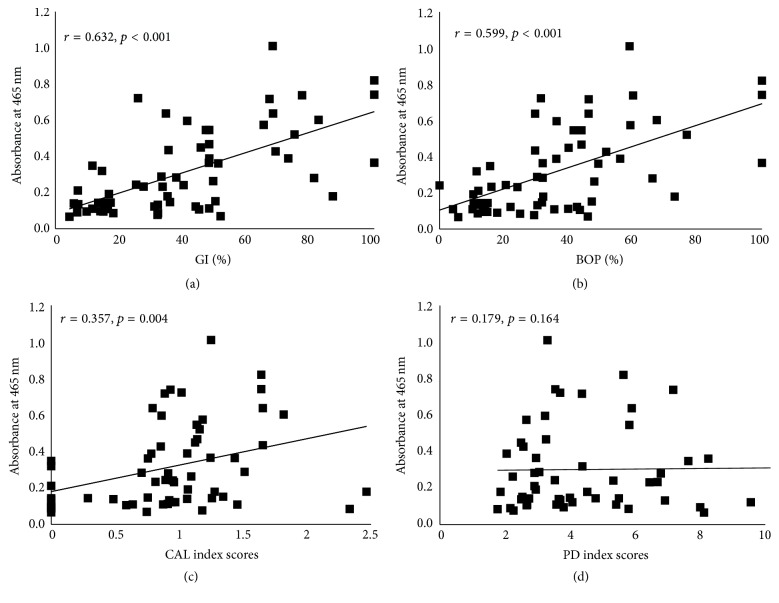
Correlations between salivary MPO activity and periodontal clinical parameters including (a) gingival bleeding index (GI), (b) bleeding on probing (BOP), (c) clinical attachment loss (CAL) index scores, and (d) pocket depth (PD) index scores. PD index score in each individual = [(1 × number of sites with PD score 1) + (2 × number of sites with PD score 2) + (3 × number of sites with PD score 3)]/total measured PD sites. CAL index score in each individual = [(1 × number of sites with CAL score 1) + (2 × number of sites with CAL score 2) + (3 × number of sites with CAL score 3)]/total measured CAL sites.

The color from the reaction between salivary MPO and DAB was mainly classified as follows: near colorless in periodontally healthy individuals; moderately brown in gingivitis patients; and intensely brown in periodontitis patients. The color change was detected by naked eye and the color intensity was detected by spectrophotometry. Comparison of salivary MPO activity of the 3 studied groups, by using one-way ANOVA analysis, gave the *p* value of 0.002. In Figure 1, pairwise comparisons by using Scheffe adjustment reveal that salivary MPO activity in periodontitis patients was significantly higher than in healthy subjects (*p* = 0.003). Difference in salivary MPO activity between gingivitis patients and control healthy subjects was observed but not significant (*p* = 0.181). When comparing MPO activity in gingivitis and periodontitis groups, the *p* value was 0.059 and the mean difference between these 2 groups was 0.148 (95% CI = [−0.004]–[0.302]) which was considered as clinical significant value. Significant correlations were observed (*p* < 0.01) between salivary MPO activity and severity of periodontal diseases as measured by the clinically periodontal parameters: GI (*r* = 0.632, *p* < 0.001), BOP (*r* = 0.599, *p* < 0.001), PD index scores (*r* = 0.179, *p* = 0.164), and CAL index scores (*r* = 0.357, *p* = 0.004) (Figure 2).  Table 2 shows the efficiency of this assay in terms of sensitivity, specificity, and predictive values. The power of differentiation between healthy subjects and periodontal disease patients was estimated at the cut-off value of 0.106 (absorbance at 465 nm) by the receiver operating characteristic curve (ROC) method. The area under ROC curve (AUC) was 0.743 (95% CI = 0.586–0.901). The MPO performance of healthy and gingivitis subjects versus periodontitis patients was also analyzed at the cut-off value of 0.365 (absorbance at 465 nm) (AUC = 0.749, 95% CI = 0.627–0.872). Multiple logistic regression was conducted to investigate the significance of MPO activity in relation to periodontal status by adjusting demographic and clinical status. For healthy versus periodontal patients, one unit increase in MPO activity resulted in an increase in odds of periodontal diseases with the estimate of 1257.68 (95% CI = 0.96 to >999, *p* = 0.051). For healthy and gingivitis subjects versus periodontitis patients, an increase in odds of periodontal diseases with the estimate of 36.23 (95% CI = 1.20–1097.66, *p* = 0.039) was obtained with each unit increase in MPO activity.

## 4. Discussion

MPO is often used as an inflammatory marker for early detection of several diseases including urinary tract infection [21], ischemic heart disease and acute coronary heart syndrome [5], and cardiovascular risk in prepubertal obese children [22] and in patients with type 2 diabetes [23]. Periodontal diseases, caused from bacterial infection followed by inflammation, may lead to tooth loss. Therefore, screening of periodontal diseases is important for early treatment. In the present study, the highest MPO activity was observed in saliva from periodontitis patients, followed by gingivitis patients and finally periodontally healthy individuals (Figure 1). MPO was detected in the saliva and GCF. Our findings are consistent with previous studies reporting increased MPO activity in periodontal disease patients [24, 25]. Utilization of saliva samples in the present study was more suitable and practical than using GCF samples in terms of a rapid sample collecting. Our results demonstrated significantly positive correlations between salivary MPO activity and clinical parameters of periodontal diseases (Figure 2). Salivary MPO activity correlated well with GI and BOP. These findings suggested that MPO activity may be a good candidate marker for detecting the occurrence of inflammation in the periodontal tissues. However, salivary MPO activity showed fair correlation with CAL and PD index scores. Our observations implied that MPO activity might not be a strong candidate marker for detecting alveolar bone loss in the periodontal tissues.

In an attempt to investigate biomarkers for periodontal diseases, the other enzymes which associated with cell injury, including creatine kinase (CK), lactate dehydrogenase (LDH), aspartate aminotransferase (AST), alanine aminotransferase (ALT), gamma glutamyl transferase (GGT), alkaline phosphatase (ALP), and acidic phosphatase (ACP), in saliva from patients with periodontal diseases (*n* = 30) and control group (*n* = 20) were studied by Todorovic et al. [26]. There was a positive correlation between the activity of these salivary enzymes and the gingival index [26]. Analysis of these enzyme activities requires an automatic analyzer, whereas MPO activity assay in the present study can be measured by simple colorimetric method. Salivary MPO activity also showed positive correlation with GI and BOP as shown in Figure 2.

Significant difference in salivary MPO activity was found between periodontitis patients and periodontally healthy individuals (*p* = 0.003) (Figure 1). The present assay did not demonstrate significant differences in salivary MPO activity between gingivitis patients and periodontally healthy individuals (*p* = 0.181) but MPO activity in gingivitis patients tended to be higher than in periodontally healthy individuals. Salivary MPO activity in gingivitis group was lower than in periodontitis group, but not statistically significant (*p* = 0.059); the mean difference (0.148) between gingivitis and periodontitis groups was considered as clinically significant value (95% CI = [−0.004]–[0.302]). Regarding diagnostic value, ROC analysis for distinguishing healthy subjects from subjects with gingivitis and periodontitis yielded an AUC of 0.743 with 94.12% sensitivity and 90.57% positive predictive value (Table 2). However, the specificity and negative predictive value were lower (54.55% and 66.67%, resp.). Hence, these data possibly reflect a considerable number of false positives. In addition, an AUC of 0.749 with 68.42% sensitivity and 81.40% specificity was obtained in differentiating of periodontitis patients from healthy and gingivitis subjects. The observation of reasonable negative predictive value (85.37%) suggests that the assay of salivary MPO activity may serve as a potential screening biomarker, rather than a diagnostic marker for differentiating periodontal patients from nonperiodontal subjects. It should be stressed that periodontal tissues in the patients with periodontitis are gradually destroyed. Even though the statistical significance was not reached, high MPO activity may indicate the risk of periodontal diseases (OR = 1257.68, 95% CI = 0.96 to >999, *p* = 0.051). By contrast, elevated MPO activity is able to predict periodontitis from healthy and gingivitis subjects (OR = 36.23, 95% CI = 1.20–1097.66, *p* = 0.039). Thus salivary MPO activity may be used as a risk indicator for periodontal diseases based on a simple screening method at least to raise awareness of deteriorating oral health, especially in the remote areas. Individuals with positive results from this screening test should be referred to dentists for a complete clinical examination of their oral health status. On this basis, the higher the sensitivity and specificity, the better the diagnostic test. Armitage et al. [27] stated that “there are, however, no preset upper and lower limits of sensitivity and specificity values that determine if a diagnostic test is clinically useful.” According to the results of the present study, to improve both sensitivity and specificity of the test, further investigation with a larger sample size is warranted. Taken together, these findings suggest that MPO activity reflects a response to inflammation in periodontal diseases and may be used as an early warning examination tool rather than a definitive diagnostic tool.

## 5. Conclusions

The present study demonstrated a significant increase in level of salivary MPO activity in periodontal patients using colorimetric method. An association between MPO activity and clinically periodontal parameters was observed. Our finding suggested that MPO may serve as a candidate marker for screening the oral health status of Thai people in rural areas.

## Figures and Tables

**Table 1:** Demographic characteristics, salivary pH, and periodontal clinical parameters of the study subjects.

Characteristics	Healthy (*n* = 11)	Gingivitis (*n* = 32)	Periodontitis (*n* = 19)
Demographic characteristics			
Age in years^*∗*^	26.72 ± 8.59	27.43 ± 10.36	49.16 ± 13.93^b,c^
Female (%)	81.81	50.00	68.42
Periodontal parameters^*∗*^			
Salivary pH	6.92 ± 0.45	6.95 ± 0.33	6.97 ± 0.36
GI (%)	11.09 ± 4.34	41.06 ± 18.36^a^	56.44 ± 29.58^b,c^
BOP (%)	12.69 ± 3.11	33.04 ± 15.96^a^	52.33 ± 28.13^b,c^
CAL index scores	0.00 ± 0.00	0.91 ± 0.25^a^	1.44 ± 0.42^b,c^
PD index scores	0.97 ± 0.11	1.06 ± 0.01^d^	1.18 ± 0.17^b,c^

GI: gingival index, BOP: bleeding on probing, CAL: clinical attachment loss, and PD: probing depth.

PD index score in each individual = [(1 × number of sites with PD score 1) + (2 × number of sites with PD score 2) + (3 × number of sites with PD score 3)]/total measured PD sites.

CAL index score in each individual = [(1 × number of sites with CAL score 1) + (2 × number of sites with CAL score 2) + (3 × number of sites with CAL score 3)]/total measured CAL sites.

^*∗*^Mean ± SD.

Statistical analysis: One-way ANOVA.

^a^Significant at *p* < 0.01 between healthy and gingivitis groups.

^b^Significant at *p* < 0.01 between healthy and periodontitis groups.

^c^Significant at *p* < 0.01 between gingivitis and periodontitis groups.

^d^Significant at *p* < 0.05 between healthy and gingivitis groups.

**Table 2:** Diagnostic efficacy of MPO assay in differentiation between healthy subjects and periodontal disease patients.

Comparative groups	ROC curve parameters		Sensitivity (%) (95% CI)	Specificity (%) (95% CI)	Predictive value (%) (95% CI)	
	Cut-off value	AUC (95% CI)			Positive	Negative
Healthy versus gingivitis and periodontitis	0.106	0.743 (0.586–0.901)	94.12 (83.76–98.77)	54.55 (23.38–83.25)	90.57 (79.34–96.87)	66.67 (29.93–92.51)

Healthy and gingivitis versus periodontitis	0.365	0.749 (0.627–0.872)	68.42 (43.45–87.42)	81.40 (66.60–91.61)	61.90 (38.44–81.89)	85.37 (70.83–94.43)

CI: confidence interval.

The cut-off value was calculated using the receiver operating characteristic curve (ROC) method.

Sensitivity, specificity, and positive and negative predictive values were analyzed by using area under ROC (AUC).

ROC plot displayed true positive sensitivity and false positive.
